# Body mass index is an independent predictor of acute kidney injury after urgent aortic arch surgery for acute DeBakey Type I aortic dissection

**DOI:** 10.1186/s13019-021-01533-8

**Published:** 2021-05-26

**Authors:** Taoshuai Liu, Yuwei Fu, Jie Liu, Yongmin Liu, Junming Zhu, Lizhong Sun, Ming Gong, Ran Dong, Hongjia Zhang

**Affiliations:** 1 Department of Cardiac Surgery, Beijing Aortic Disease Center, Beijing Anzhen Hospital, Capital Medical University, Beijing Institute of Heart Lung and Blood Vessel Diseases and Beijing Engineering Research Center of Vascular Prostheses, No.2 Anzhen Street, Beijing, 100029 China; 2 Department of Ultrasound, Peking University International Hospital, No. 1, Shengmingyuan Road, Zhongguancun Life Science Park, Changping District, Beijing, 102206 China; 3 Department of Vascular and Endovascular Surgery, Chinese PLA General Hospital, Beijing, 100853 China

**Keywords:** Acute kidney injury, Aortic total arch replacement surgery, Body mass index

## Abstract

**Background:**

Aortic arch surgery and obesity are both related to the risk of acute kidney injury. Our hypothesis was that the risk of postoperative acute kidney injury increases as body mass index increases in patients undergoing urgent aortic total arch replacement surgery for acute DeBakey Type I aortic dissection.

**Methods:**

We conducted a retrospective cohort study in Beijing Anzhen Hospital from December 2015 to April 2017. All patients receiving urgent aortic total arch replacement surgery with a frozen elephant trunk implant for acute DeBakey Type I aortic dissection were included. Body mass index was calculated based on height and weight. Acute kidney injury was diagnosed based on the Kidney Disease Improving Global Outcomes standards.

**Results:**

We included 115 consecutive patients in this study. A total of 53.0% (*n* = 61) of patients had acute kidney injury. The mean age was 47.8 ± 10.7 years, and 25.2% were women. Mean body mass index was 26.2 ± 3.9 kg/m^2^. The results of a univariate analysis showed that BMI, eGFR, CPB time, operative time, intraoperative blood loss, intraoperative amount of PRBCs, and respiratory failure were significantly correlated with AKI. In-hospital mortality was obviously increased in the acute kidney injury group (13.1% vs 1.9%; *P* = 0.025). Multivariate logistic regression showed that body mass index was associated with postoperative acute kidney injury after adjusting for other confounding factors (odds ratio = 1.16; 95% confidence interval: 1.02–1.33; *P* = 0.0288). The risk of postoperative AKI in the BMI ≥ 24 kg/m^2^ group was increased by 2.35 times (OR = 3.35, 95% CI: 1.15–9.74; *p* = 0.0263).

**Conclusions:**

Body mass index was an independent predictor of acute kidney injury after urgent aortic total arch replacement surgery with a frozen elephant trunk implant.

## Background

Acute kidney injury (AKI) is not an uncommon complication following cardiovascular surgery. The incidence of AKE is approximately 30%, and it markedly increases mortality and morbidity [1–4]. The occurrence of AKI following cardiac surgery is influenced by many factors, including perioperative red blood cell transfusions, preoperative anemia, increased age, and intraoperative cardiopulmonary bypass (CPB) time [5–7]. However, AKI after aortic surgery has not been extensively studied. This kind of operation can potentially increase the likelihood of developing AKI due to the operative complexity, including a longer CPB time and circulatory arrest.

Currently, obesity is a worldwide public health issue. It is correlated with a broad range of cardiovascular disorders [8], including hypertension, insulin resistance, and diabetes mellitus [9]. Obese patients in general are prone to AKI owing to the higher burden of comorbid diseases and potential structural changes in the kidneys, even though their serum biochemistry is normal [10, 11]. Few studies have examined the effect of obesity on AKI in patients undergoing urgent aortic total arch replacement (TAR) surgery with a frozen elephant trunk implant (FET) for acute DeBakey Type I aortic dissection (ADTIAD).

Therefore, our objective was to investigate the effect of body mass index (BMI) on AKI in patients who underwent urgent TAR+FET after adjusting for all known confounding factors. Our hypothesis was that the risk of AKI would increase as BMI increased.

## Methods

### Participants

This retrospective cohort study was conducted at Beijing Anzhen Hospital in China from December 2015 to April 2017. This study was allowed by the human research and development committees of this hospital and adhered to the rules of the Declaration of Helsinki and principles of Good Clinical Practice. Individual consent was waived for the retrospective study. All patients with ADTIAD who underwent urgent TAR+FET surgery in this timeframe were included. All surgical treatments were performed by the same surgical team.

**Fig. 1:**
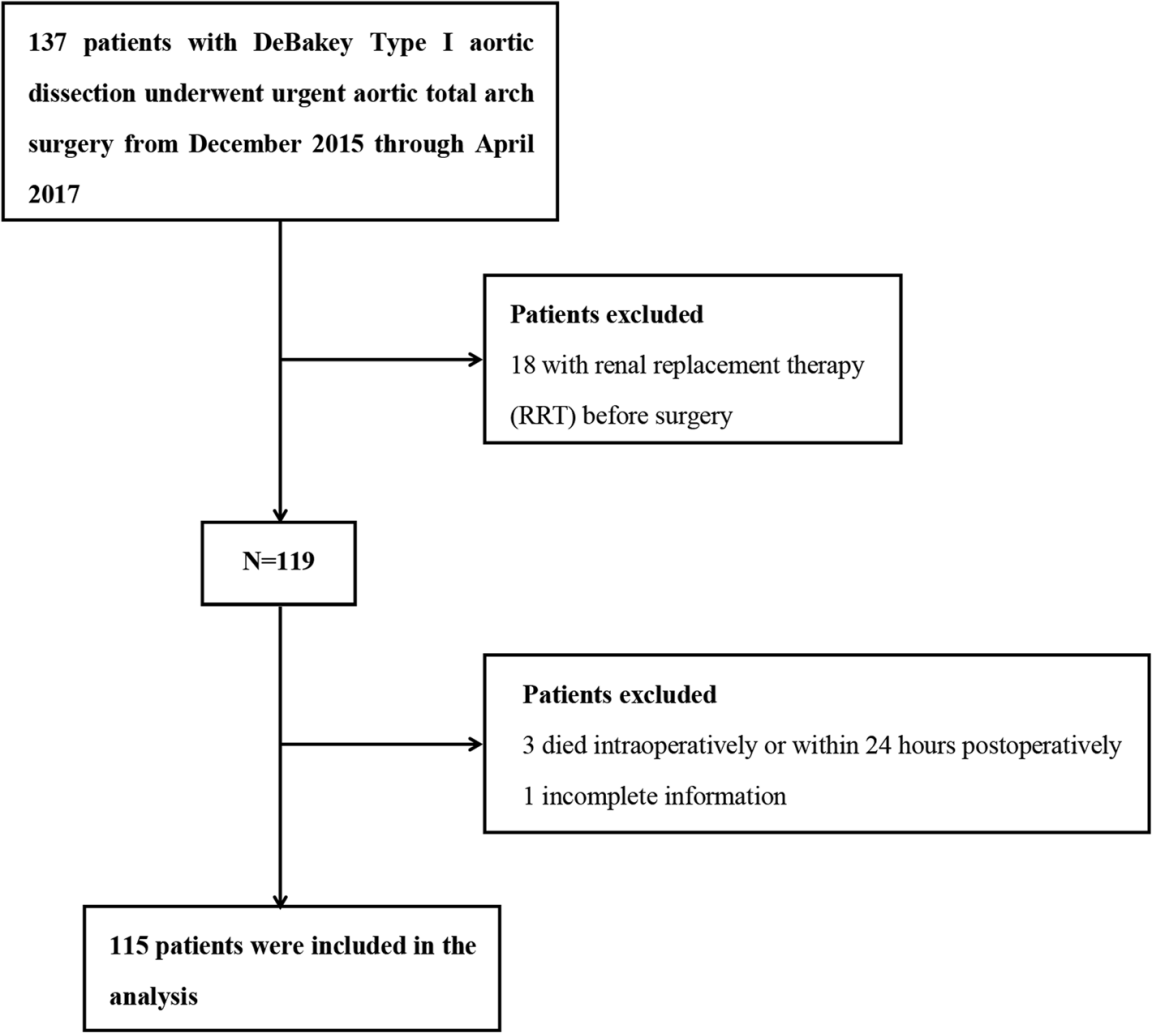
Flow diagram of the screening and enrollment of study patients

A total of 137 patients with ADTIAD undergoing urgent TAR+FET with CPB were admitted. A total of 18 patients were excluded because they underwent renal replacement therapy (RRT) before the operation. Three patients were excluded because they died intraoperatively or within 24 h after surgery. One patient who lacked serum creatinine (sCr) was also excluded. Consequently, 115 consecutive patients were involved in the final analysis. A flow chart of the screening and registration of study patients is shown in Fig. 1.

### Data collection

Trained staff collected detailed patient data from electronic medical records. Baseline characteristics included age, sex, height, weight, BMI (calculated based on height and weight), drinking history, and smoking history. Comorbidities included diabetes mellitus, hypertension, previous cerebrovascular disease, time from onset to treatment, left ventricular ejection fraction (LVEF), coronary artery disease, hematocrit, and preoperative blood urea nitrogen (BUN). Also included were data on sCr, preoperative hemoglobin, hemopericardium, eGFR (estimated glomerular filtration rate, calculated based on Epidemiology Collaboration equation), renal artery dissection, Penn class (Class Aa and Non class Aa), kidney malperfusion, acute myocardial infarction (AMI), and preoperative shock. Intraoperative data included intraoperative administration of packed red blood cells (PRBCs), intraoperative administration of plasma, intraoperative blood loss, CPB time, aortic occlusion clamp time, time of circulatory arrest, rectal temperature and nasopharyngeal temperature at circulatory arrest, type of surgery (Bentall +TAR+FET or ascending aorta replacement +TAR+FET), and combination with coronary artery bypass grafting (CABG) or with ascending aorta to femoral artery bypass surgery (aortic bypass surgery). Postoperative data included postoperative respiratory insufficiency, reoperation for bleeding, postoperative dialysis, intensive care unit (ICU) length-of-stay, hospital length-of-stay, and in-hospital death. Primary indications for renal replacement therapy were uremica, anuresis, volume overload, and obvious biochemical abnormalities.

### Outcome variables

The main endpoint event was the occurrence of AKI following urgent TAR+FET operation. Recently, Kidney Disease Improving Global Outcome (KDIGO) proposed a new range of guidelines for the characterization of AKI based on two previous classifications, RIFLE and AKIN [12, 13]. In this study, AKI was diagnosed based on the KDIGO criteria: the postoperative sCr levels increased by more than 50% within 7 days or 0.3 mg/dL within 2 days by baseline after the operation. The last sCr value measured before surgery was regarded as baseline sCr.

### Assessment of covariates

Our covariates were selected based on our prior work and studies, and from other studies examining risk factors for postoperative AKI. Hypertension, DM, preoperative hemoglobin, hematocrit, preoperative baseline sCr, preoperative BUN, CPB time, intraoperative blood loss, and intraoperative amount of PRBCs were recorded for all participants. Preoperative hemoglobin, hematocrit, preoperative baseline creatinine, and preoperative BUN were determined from the results of the laboratory test after hospital admission before surgery.

### Surgical technique

All patients received TAR with FET. This method has been reported at great length by our research team [14]. Briefly, we used the right axillary artery and atrium dextrum to perform CPB following heparinization [maintaining the activated clotting time exceeding 480 s]. The flow rates of CPB were 2.5 L/(min·m^2^). We kept the mean arterial pressure between 50 and 70 mmHg. The decision whether to perform aortic valve replacement depended on the condition of the aortic valve. If the classification of aortic regurgitation was moderate or severe, it was preferred to perform the Bentall procedure (aortic valve replacement combined with ascending aorta replacement). If there was only mild regurgitation, it was preferred to perform ascending aorta replacement only. A frozen elephant stent (MicroPort Medical Company Limited, Shanghai, China) and a four-branched artificial vessel (Maquet Cardiovascular, Wayne, NJ) were employed in this implantation. Surgery was usually performed with moderate hypothermic circulatory arrest and antegrade cerebral perfusion.

### Data analysis

Means or medians were used to express continuous variables according to the data distribution. Categorical variables are presented as percentages (%). If continuous variables were normally distributed, t-tests were used to compare groups. If the data were skewed, nonparametric Mann–Whitney U tests were applied. The predictors of AKI were recognized by univariate regression analysis. Multiple regression models were used to evaluate the effect of BMI on postoperative AKI. Both nonadjusted and adjusted models (sex; age; previous cerebrovascular disease; smoking history; renal artery dissection; eGFR; AMI; respiratory failure; CPB time; intraoperative blood loss; reoperation for bleeding; intraoperative amount of PRBCs) were applied. Whether the concomitant variable was adjusted according to the following principle: if the matched odds ratio was changed at least 10% [15] by the variable when added to this model, then an adjustment was made. A generalized additive model (GAM) was also applied to discover linear relationships. BMI was analyzed as a continuous variant in multivariable logistic models. Further analysis was performed using 24 kg/m^2^ as a cutoff value based on the Chinese population BMI classification criteria [16]. Interaction and stratified analyses were performed based on age (< 60 and ≥ 60 years), sex, hypertension, smoking history, drinking history, coronary artery disease, previous cerebrovascular disease, CPB time (< 204 or ≥ 204 min), aortic cross clamp time (< 115 or ≥ 115 min), circulation arrest time (< 27 or ≥ 27 min), operation time (< 8 or ≥ 8 h), intraoperative blood loss (< 1500 or ≥ 1500 mL), intraoperative amount of PRBCs (< 2 or ≥ 2 U), intraoperative amount of plasma (< 400 or ≥ 400 mL), and hemoglobin (< 135 or ≥ 135 g/L). All of the analyses were conducted by the statistical software packages R and EmpowerStats.

## Results

### Baseline characteristics

After the exclusion criteria were applied, 115 consecutive patients were admitted to this cohort, with a median age of 47.8 ± 10.7 years; 29 (25.2%) were female. There were 61 patients (53%) with AKI. The average BMI was 26.2 ± 3.9 kg/m^2^. The average preoperative BUN was 7.2 ± 2.5 mmol/L, and the preoperative sCr was 86.2 ± 29.1 μmol/L. Comorbidities included hypertension (80%), diabetes mellitus (6.1%), previous cerebrovascular disease (5.2%), and coronary artery disease (5.2%). In total, 20% of patients required RRT. The baseline characteristics of the 115 study patients with AKI and non-AKI are given in Table 1.

**Table 1:** Characteristics of the study patients between AKI and No-AKI

Variable	No-AKI (***n*** = 54)	AKI (***n*** = 61)	***P***-value
Age	46.8 ± 11.0	48.7 ± 10.4	0.342
Sex			0.486
Male	42 (77.8%)	44 (72.1%)	
Female	12 (22.2%)	17 (27.9%)	
**BMI (kg/m**^**2**^**)**	**25.0 ± 3.8**	**27.2 ± 3.7**	**0.002**
Diabetes mellitus	1 (1.9%)	6 (9.8%)	0.074
Hypertension	42 (77.8%)	50 (82.0%)	0.575
Previous cerebrovascular disease	4 (7.4%)	2 (3.3%)	0.320
Coronary artery disease	3 (5.6%)	3 (4.9%)	0.878
Smoking history	22 (40.7%)	34 (55.7%)	0.108
Drinking history	11 (20.4%)	12 (19.7%)	0.926
Time from onset to treatment(h)	60 (24–138)	24 (20–48)	0.005
Renal artery dissection	10 (18.5%)	7 (11.5%)	0.288
Hemopericardium	9 (16.7%)	10 (16.4%)	0.969
Penn class			0.665
Class Aa	34 (63.0%)	36 (59.0%)	
Non class Aa	20 (37.0%)	25 (41.0%)	
Kindey malperfusion	3 (5.6%)	4 (6.6%)	0.823
AMI	2 (3.7%)	7 (11.5%)	0.121
Preoperative shock	9 (16.7%)	10 (16.4%)	0.969
Preoperative sCr (umol/L)	82.0 ± 30.0	90.0 ± 27.9	0.141
BUN (mmol/L)	7.1 ± 2.7	7.3 ± 2.4	0.724
Hemoglobin (g/L)	137.2 ± 18.6	135.9 ± 16.3	0.689
Hematocrit (%)	39.7 ± 5.0	39.1 ± 4.2	0.443
**eGFR mL/(min·1.73 m**^**2**^**)**	**94.2 ± 21.2**	**83.5 ± 22.8**	**0.011**
**CPB time (min)**	**199.1 ± 45.6**	**222.0 ± 62.0**	**0.027**
Aortic cross clamp time (min)	120.2 ± 49.0	127.0 ± 36.1	0.400
Circulatory arrest time (min)	27.4 ± 9.6	27.5 ± 7.6	0.914
Operation time (h)	23.1 ± 1.6	22.7 ± 1.5	0.142
Nasopharyngeal temperature at circulatory arrest (°C)	25.5 ± 2.3	25.1 ± 2.0	0.358
**Rectal temperature at circulatory arrest (°C)**	**27.9 ± 1.6**	**28.8 ± 2.0**	**0.007**
**Intraoperative amount of PRBCs (mL)**	**300.0 (0.0–600.0)**	**600.0 (0.0–600.0)**	**0.043**
**Intraoperative blood loss (mL)**	**1300.0 (1000.0–1575.0)**	**1500.0 (1200.0–2000.0)**	**0.024**
Intraoperative amount of plasma (mL)	400.0 (0.0–550.0)	400.0 (0.0–800.0)	0.102
Combined with CABG	3 (5.6%)	5 (8.2%)	0.578
Combined with aortic bypass surgery	0 (0.0%)	1 (1.6%)	0.345
Bentall+TAR+FET	22 (40.7%)	25 (41.0%)	0.979
**Respiratory failure**	**2 (3.70%)**	**18 (29.51%)**	**< 0.001**
**Length of ICU**	**1.9 (1.0–3.0)**	**4.0 (2.0–9.0)**	**< 0.001**
**Length of in hospital**	**1 (1.9%)**	**8 (13.1%)**	**0.025**
**Reoperation for bleeding**	**1 (1.9%)**	**8 (13.1%)**	**0.025**
**In-hospital mortality**	**1 (1.9%)**	**8 (13.1%)**	**0.025**

Bold value indicates significance at *p* < 0.05. Results are expressed as n (%) or mean ± SD or median [IQR]

*AKI* Acute Kidney Injury, *AMI* Acute Myocardial Infarction, *BMI* Body Mass Index, *BUN* Blood Urea Nitrogen, *CPB* Cardiopulmonary Bypass, *CABG* Coronary Artery Bypass Grafting, *eGFR* estimated Glomerular Filtration Rate, *FET* Frozen Elephant Trunk, *ICU* Intensive Care Unit, *PRBCs* Packed Red Blood Cells, *sCr* serum Creatinine, *SD* Standard Deviation, *TAR* Total Arch Replacement, *IQR* Interquartile Range

### Univariate analysis of predictors of AKI

The results of a univariate analysis showed that BMI, eGFR, CPB time, operative time, intraoperative blood loss, intraoperative amount of PRBCs and respiratory failure were significantly correlated with AKI. Smoking, hemoglobin levels, hypertension, hematocrit, preoperative sCr, BUN, aortic cross clamp time, intraoperative amounts of plasma and circulatory arrest time were not significantly associated with AKI. The results are given in Table 2.

**Table 2:** Univariate analysis of risk factors associated with postoperative AKI in patients with ADTIAD

Variable	Statistics	OR (95%CI)	***P***-value
Age	47.81 + 10.70	1.02 (0.98, 1.05)	0.339
Sex			
Male	86 (74.78%)	1.0	
Female	29 (25.22%)	1.35 (0.58, 3.17)	0.487
**BMI (kg/m**^**2**^**)**	**26.2 + 3.9**	**1.18 (1.06, 1.33)**	**0.003**
Diabetes mellitus	7 (6.09%)	5.78 (0.67, 49.62)	0.110
Hypertension	92 (80.00%)	1.30 (0.52, 3.24)	0.576
Previous cerebrovascular disease	6 (5.22%)	0.42 (0.07, 2.41)	0.333
Coronary artery disease	6 (5.22%)	0.88 (0.17, 4.55)	0.878
Smoking history	56 (48.70%)	1.83 (0.87, 3.85)	0.110
Drinking history	23 (20.00%)	0.96 (0.38, 2.39)	0.926
Renal artery dissection	17 (14.78%)	0.57 (0.20, 1.62)	0.292
Hemopericardium	19 (16.52%)	0.98 (0.37, 2.63)	0.969
Penn class			
Class Aa	70 (60.87%)	1.0	
Non class Aa	45 (39.13%)	1.18 (0.56, 2.50)	0.665
Kidney malperfusion	7 (6.09%)	1.19 (0.25, 5.59)	0.823
AMI	9 (7.83%)	3.37 (0.67, 16.98)	0.141
Preoperative shock	19 (16.52%)	0.98 (0.37, 2.63)	0.969
Preoperative sCr (umol/L)	86.22 + 29.07	1.01 (1.00, 1.02)	0.147
BUN (mmol/L)	7.21 + 2.51	1.03 (0.89, 1.19)	0.722
Hemoglobin (g/L)	136.53 + 17.32	1.00 (0.97, 1.02)	0.686
Hematocrit (%)	39.38 + 4.61	0.97 (0.89, 1.05)	0.440
**eGFR mL/(min·1.73m**^**2**^**)**	**88.55 + 22.64**	**0.98 (0.96, 1.00)**	**0.013**
**CPB time (min)**	**211.25 + 55.90**	**1.01 (1.00, 1.02)**	**0.032**
Aortic cross clamp time (min)	123.81 + 42.57	1.00 (0.99, 1.01)	0.401
Circulatory arrest time (min)	27.44 + 8.54	1.00 (0.96, 1.05)	0.913
**Operation time (h)**	**8.36 + 1.89**	**1.33 (1.07, 1.65)**	**0.009**
Nasopharyngeal temperature at circulatory arrest (°C)	22.86 + 1.55	0.83 (0.65, 1.06)	0.143
Rectal temperature at circulatory arrest (°C)	25.29 + 2.13	0.92 (0.77, 1.10)	0.356
Combined with CABG	8 (6.96%)	1.52 (0.35, 6.67)	0.581
Combined with aortic bypass surgery	1 (0.87%)	§	0.992
Bentall+TAR+FET	47 (40.87%)	1.01 (0.48, 2.13)	0.979
**Respiratory failure**	**18 (29.51%)**	**10.88 (2.39, 49.55)**	**0.0020**
**Intraoperative amount of PRBCs (mL)**	**393.91 + 413.17**	**1.00 (1.00, 1.00)**	**0.048**
**Intraoperative blood loss (mL)**	**1487.8 ± 710.5**	**1.00 (1.00, 1.00)**	**0.032**
Intraoperative amount of plasma (mL)	400 (0–600)	1.00 (1.00, 1.00)	0.104
Reoperation for bleeding	9 (7.83%)	8.00 (0.97, 66.21)	0.054

Bold value indicates significance at p < 0.05. § = The result failed because of the small sample size

Results are expressed as n (%) or mean ± SD or median [IQR]

*AKI* Acute Kidney Injury, *AMI* Acute Myocardial Infarction, *BMI* Body Mass Index, *BUN* Blood Urea Nitrogen, *CPB* Cardiopulmonary Bypass, *CABG* Coronary Artery Bypass Grafting, *eGFR* estimated Glomerular Filtration Rate, *FET* Frozen Elephant Trunk, *ICU* Intensive Care Unit, *PRBCs* Packed Red Blood Cells, *sCr* serum Creatinine, *SD* Standard Deviation, *TAR* Total Arch Replacement, *IQR* Interquartile Range

### The linear relationship between BMI and AKI after adjusting for covariates

**Fig. 2:**
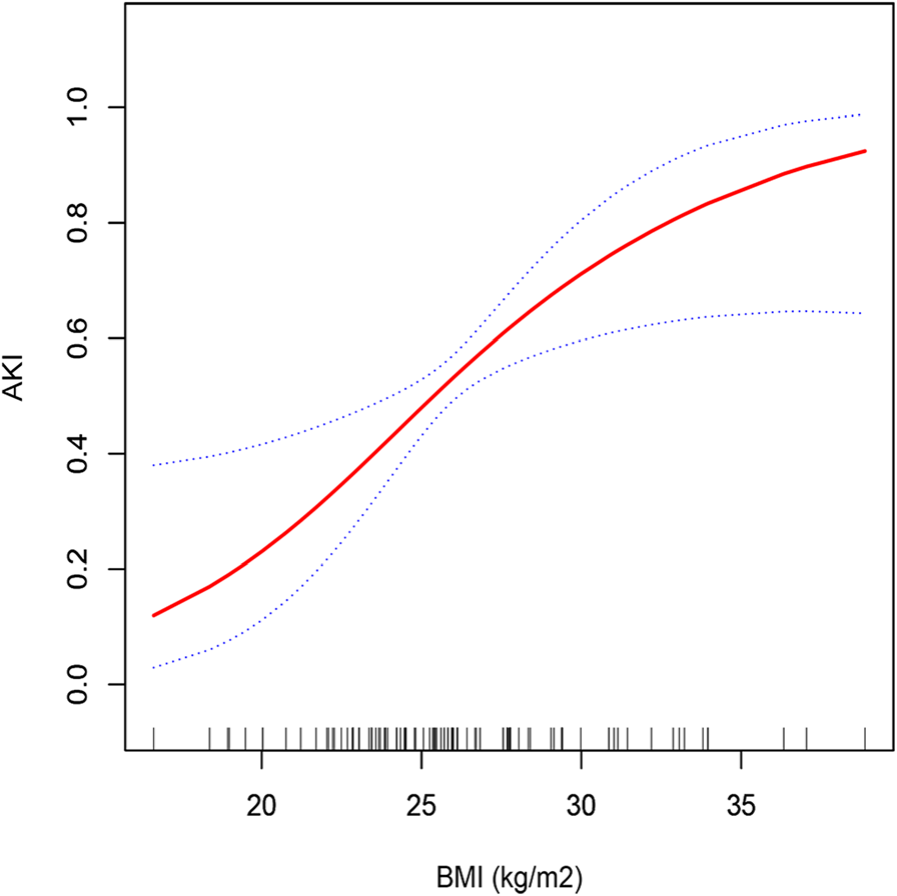
A linear relationship between BMI and postoperative AKI was observed after adjusting for age, sex, previous cerebrovascular disease, smoking history, renal artery dissection, eGFR, and AMI. In the figure, the red line indicates the estimated risk of acute kidney injury, and the blue points represent the pointwise 95% confidence interval

Spline smoothing was implemented using a GAM to identify the linear relationship between BMI and AKI after adjusting for age, sex, previous cerebrovascular disease, smoking history, renal artery dissection, eGFR, and AMI. The red line represents the fitting spline. The blue points represent the 95% confidence intervals. The result is shown in Fig. 2.

### Multivariate analysis: independent predictor of AKI

Table 3 reveals the consequences of multivariate regression analysis. We constructed three models: (I) unadjusted; (II) adjusted for age and sex; (III) adjusted for age, sex, previous cerebrovascular disease, smoking history, renal artery dissection, eGFR, AMI, respiratory failure, CPB time, intraoperative blood loss, reoperation for bleeding, and intraoperative amount of PRBCs. Model I showed a significant association between BMI and AKI [odds ratio (OR) = 1.18; 95% confidence interval (CI): 1.06–1.33; *p* = 0.003]. In model II (adjusted for age and sex), the results were also significant (OR = 1.21, 95% CI: 1.08–1.36; *p* = 0.002). In model III (adjusted for age; sex; previous cerebrovascular disease; smoking history; renal artery dissection; eGFR; AMI; respiratory failure; CPB time; intraoperative blood loss; reoperation for bleeding; intraoperative amount of PRBCs), the results were still significant (OR = 1.16, 95% CI: 1.02–1.33; *p* = 0.0288).

**Table 3:** Multivariable analysis to assess the independent impact of BMI on postoperative AKI in patients with ADTIAD using non-adjusted and adjusted logistic regression model

Variable	Model I		Model II		Model III	
	OR (95%CI)	***P***-value	OR (95%CI)	***P***-value	OR (95%CI)	***P***-value
BMI (kg/m^2^)	1.18 (1.06, 1.33)	0.003	1.21(1.08, 1.36)	0.002	1.16 (1.02, 1.33)	0.0288
BMI groups (kg/m^2^)						
< 24	1.0		1.0		1.0	
≧24	3.27 (1.43, 7.48)	0.005	4.02 (1.64, 9.89)	0.002	3.35 (1.15, 9.74)	0.0263

*ADTIAD* Acute DeBakey Type I Aortic Dissection, *AKI* Acute Kidney Injury, *AMI* Acute Myocardial Infarction, *BMI* Body Mass Index, *eGFR* estimated Glomerular Filtration Rate, *OR* Odd Ratio, *95% CI* 95% Confidence Interval, *CPB time* Cardiopulmonary Bypass time, *PRBCs* Packed Red Blood Cells

Model I: adjust for none

Model II: adjust for age; sex

Model III: adjust for age; sex; previous cerebrovascular disease; smoking history; renal artery dissection; eGFR; AMI; respiratory failure; CPB time; intraoperative blood loss; reoperation for bleeding; intraoperative amount of PRBCs

Secondary analyses were performed using 24 kg/m^2^ as a cutoff value based on the Chinese BMI classification reference standard. In the nonadjusted model, BMI < 24 kg/m^2^ was taken as a reference. The risk of postoperative AKI in the BMI ≥24 kg/m^2^ group increased by 2.27 times (OR = 3.27, 95% CI: 1.43–7.48; *p* = 0.005). After adjusting for other confounding factors, the results were still statistically significant; the risk of AKI increased by 2.35 (OR = 3.35, 95% CI: 1.15–9.74; *p* = 0.0263).

### Stratified analysis

**Fig. 3:**
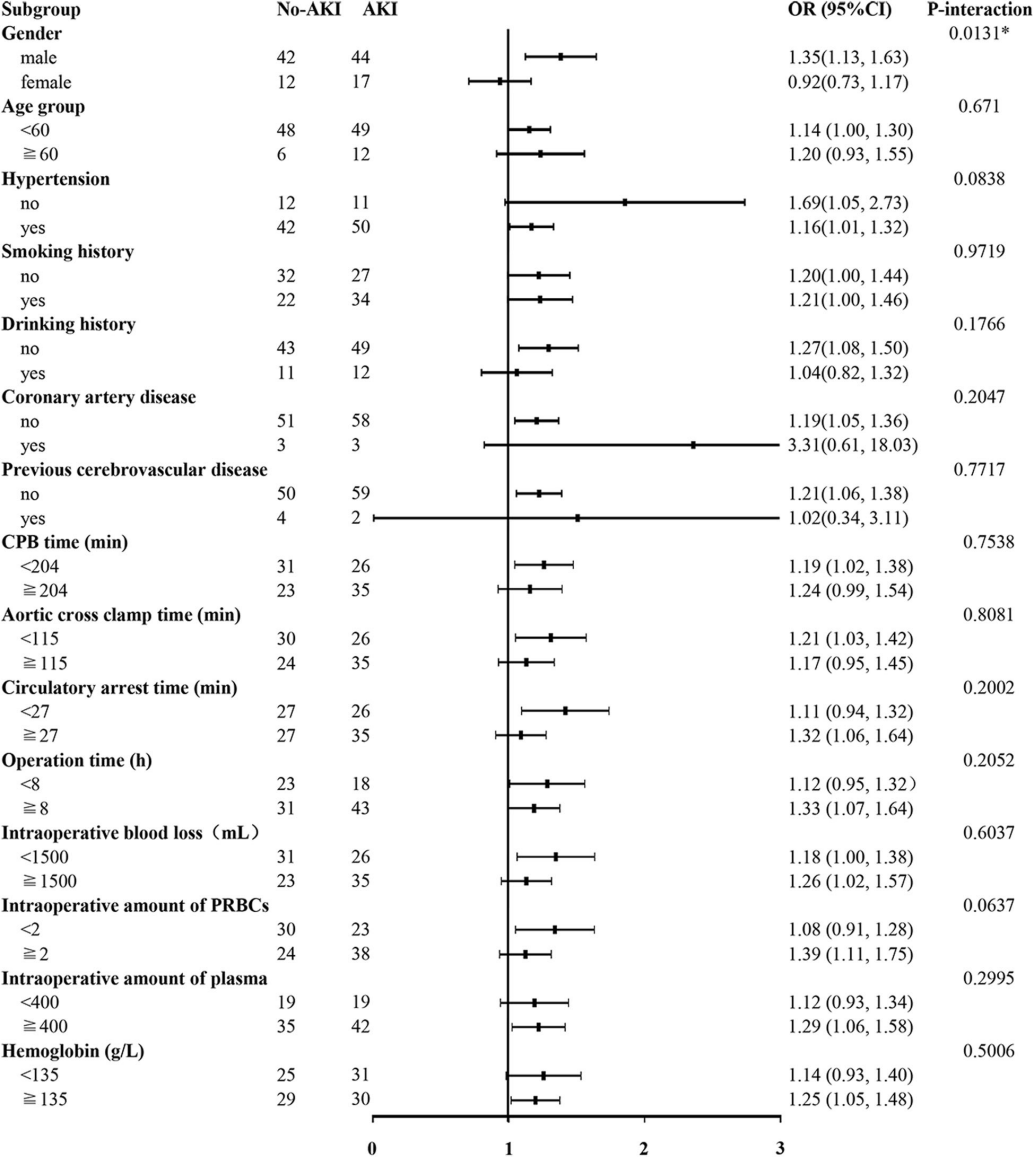
Subgroup analysis of the association between BMI and AKI in patients with ADTIAD. Each stratification adjusted for all the factors (age, diabetes mellitus, hypertension, previous cerebrovascular disease, coronary artery disease, smoking history, drinking history, coronary artery disease, previous cerebrovascular disease, CPB time, aortic cross clamp time, circulation arrest time, operation time, intraoperative blood loss, intraoperative amount of PRBCs, and intraoperative amount of plasma and hemoglobin), with the exception of the stratification factor itself

Stratified analysis was performed in patients categorized by age (age < 60 years and age ≥ 60 years), diabetes mellitus, hypertension, previous cerebrovascular disease, coronary artery disease, smoking history, drinking history, coronary artery disease, previous cerebrovascular disease, CPB time (< 204 or ≥ 204 min), aortic cross clamp time (< 115 or ≥ 115 min), circulation arrest time (< 27 or ≥ 27 min), operation time (< 8 or ≥ 8 h), intraoperative blood loss (< 1500 or ≥ 1500 mL), intraoperative amount of PRBCs (< 2 or ≥ 2 U), intraoperative amount of plasma (< 400 or ≥ 400 mL), and hemoglobin (< 135 or ≥ 135 g/L). BMI was still an independent predictor of AKI in these high-risk subgroups. The results of the stratified analysis are given in Fig. 3.

## Discussion

In this cohort study of 115 patients who underwent urgent TAR+FET for ADTIAD, we found that BMI was positively correlated with postoperative AKI. A 1-kg/m^2^ increase in BMI was correlated with an 18% higher risk of postoperative AKI. Taking BMI < 24 kg/m^2^ as a reference, the risk of AKI in the BMI ≥24 kg/m^2^ group increased by 2.35 times. This confirms the relationship between BMI and postoperative AKI and verifies our hypothesis.

This finding was the same as the results of Kumar et al. [17], who identified an obvious increase in the incidence of postoperative AKI in patients with BMI greater than 40 kg/m^2^. Certainly, there were some obvious differences between their study and ours. First, many patients were obese in their study cohort (46%, vs 13% in the current study). Second, there were no patients in our study with a BMI equal to or exceeding 40 kg/m^2^. Third, they did not examine the impact of baseline hemoglobin and hematocrit on AKI, which is an independent risk factor associated with AKI [18]. O’Sulliva et al. [19] analyzed 432 consecutive patients who received cardiovascular surgery with CPB and found that a BMI of 30 kg/m^2^ or greater was an independent predictor of AKI (OR = 2.12, 95% CI: 1.27–3.54; *p* = 0.004). This finding was consistent with the results of our study. Moreover, several other studies [20–24] have found that BMI was an independent predictor of the development of AKI in patients who received cardiovascular surgery, which further confirmed our findings.

Previous studies have shown varying results. Roh et al. [25] analyzed 98 patients who underwent thoracic aorta replacement due to AD and found that BMI was not correlated with AKI (OR = 1.04, 95% CI: 0.93–1.16; *p* = 0.54). However, as the authors acknowledged, the smaller number of participants might have led to insufficient statistical validity. Vellinga et al. [26] analyzed 565 consecutive patients undergoing CABG with the employment of cardiopulmonary bypass and did not find that BMI was associated with postoperative AKI. This could be because the study population was different.

The potential molecular mechanisms for the connection between BMI and AKI remain unclear. Billings et al. [27] explored the connection in 445 consecutive patients who underwent cardiac surgery. The results showed that BMI was independently associated with AKI. Additionally, baseline F2-isoprostane, intraoperative F2-isoprostane, and intraoperative plasminogen activator inhibitor-1 concentrations were also independent predictors of AKI. However, after adjusting for these variables, BMI was no longer associated with AKI, indicating that the influence may be interrupted by oxidative stress.

This study has several strengths. First, the patients selected for this study comprised a homogeneous population of patients with ADTIAD who underwent urgent TAR+FET. Second, we adopted the KDIGO guidelines for AKI instead of the previous 2 classifications, as the KDIGO guidelines have been revised more recently and are clear and simple for clinical use. Third, although the study was observational research with possible confounding variables, we used rigorous statistical methods to minimize the remaining confounders. Finally, we handled target independent variables as both continuous variables and categorical variables. Such an approach can reduce the contingency in the data analysis and enhance the robustness of the results.

It was vital to explore the predictors related to AKI. Emphasis on obesity as a problem may be helpful for clinicians to deal with patients pre- and postoperatively. For elective surgical patients, weight loss may be a useful preoperative method for reducing the risk of AKI. However, it may not be applicable for emergent surgery. Furthermore, we should pay more attention to patients with higher BMIs who are undergoing urgent TAR+FET to monitor them for postoperative AKI.

There were also some limitations to the present study: (1) in this study, our research subjects were patients who received aortic surgery for ADTIAD. Therefore, there is a certain deficiency in the universality and extrapolability of the research. (2) Because we excluded patients requiring RRT before surgery, the findings of this study cannot be used for this group of patients. (3) This was a retrospective cohort study, which may have limited the observational study design.

## Conclusion

BMI was an independent predictor of AKI in patients who underwent urgent TAR+FET surgery for ADTIAD. It was crucial to explore the molecular mechanisms for performing preventative therapies.

## Data Availability

The datasets used and/or analysed during the current study are available from the corresponding author on reasonable request.

## Abbreviations

ADTIAD: Acute DeBakey Type I aortic dissection
AKI: Acute kidney injury
AKIN: Acute Kidney Injury Network
AMI: Acute myocardial infarction
BMI: Body mass index
BUN: Blood urea nitrogen
CABG: Coronary artery bypass grafting
CI: Confidence interval
CPB: Cardiopulmonary bypass
GAM: Generalized additive model
ICU: Intensive care unit
KDIGO: Kidney Disease Improving Global Outcomes
LVEF: Left ventricular ejection fraction
OR: Odds ratio
PRBCs: Packed red blood cells
RIFLE: Risk, Injury, Failure, Loss and End-stage
RRT: Renal replacement therapy
SCP: Selective cerebral perfusion
sCr: Serum creatinine
